# 6-Formyl-2-naphthyl *cis*-1,5,7-trimethyl-2,4-dioxo-3-aza­bicyclo­[3.3.1]nonane-7-carboxyl­ate

**DOI:** 10.1107/S1600536809054786

**Published:** 2009-12-24

**Authors:** Sheng-guang Xu, Chun-hua Wang, Jun-shen Liu, Xi-guang Liu, Long Li

**Affiliations:** a School of Chemistry & Materials Science, Ludong University, Yantai of Shandong, People’s Republic of China

## Abstract

In the title compound, C_23_H_23_NO_5_, the C_5_N ring adopts an envelope conformation with a C atom as the flap, whilst the saturated C_6_ ring fused to it adopts a chair conformation. In the crystal, inversion dimers linked by pairs of N—H⋯O hydrogen bonds generate *R*_2_^2^(8) loops.

## Related literature

For further information on the title compound as a building block in a dynamic combinatorial library, see: Xu & Giuseppone (2008 ▶). For further synthetic details, see: Askew *et al.* (1989 ▶); Rebek *et al.* (1987 ▶); Steglich & Höfle (1969 ▶); Williams *et al.* (1989 ▶); Chamontin *et al.* (1999 ▶).
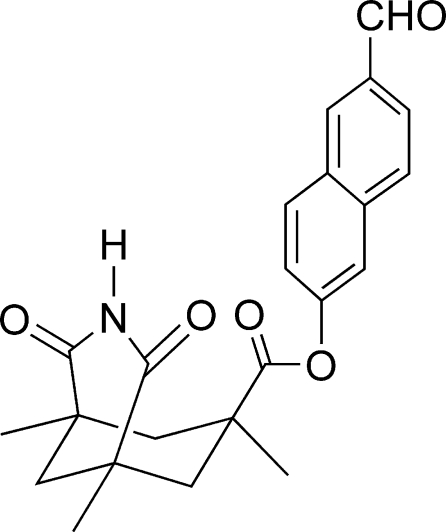


## Experimental

### 

#### Crystal data

C_23_H_23_NO_5_
*M* *_r_* = 393.42
Monoclinic, 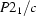

*a* = 11.896 (2) Å
*b* = 11.692 (2) Å
*c* = 16.636 (2) Å
β = 121.124 (9)°
*V* = 1980.8 (5) Å^3^
*Z* = 4
Mo *K*α radiation
μ = 0.09 mm^−1^
*T* = 298 K
0.28 × 0.20 × 0.20 mm

#### Data collection

Bruker APEXII CCD diffractometer
Absorption correction: multi-scan (*SADABS*; Bruker, 2004 ▶) *T*_min_ = 0.974, *T*_max_ = 0.982
10443 measured reflections
3897 independent reflections
3217 reflections with *I* > 2σ(*I*)
*R*_int_ = 0.018

#### Refinement

*R*[*F* ^2^ > 2σ(*F* ^2^)] = 0.038
*wR*(*F* ^2^) = 0.105
*S* = 1.03
3897 reflections
265 parameters
H-atom parameters constrained
Δρ_max_ = 0.19 e Å^−3^
Δρ_min_ = −0.22 e Å^−3^

### 

Data collection: *APEX2* (Bruker, 2004 ▶); cell refinement: *SAINT* (Bruker, 2004 ▶); data reduction: *SAINT*; program(s) used to solve structure: *SHELXS97* (Sheldrick, 2008 ▶); program(s) used to refine structure: *SHELXL97* (Sheldrick, 2008 ▶); molecular graphics: *XP* in *SHELXTL* (Sheldrick, 2008 ▶); software used to prepare material for publication: *SHELXL97*.

## Supplementary Material

Crystal structure: contains datablocks I, global. DOI: 10.1107/S1600536809054786/hb5273sup1.cif

Structure factors: contains datablocks I. DOI: 10.1107/S1600536809054786/hb5273Isup2.hkl

Additional supplementary materials:  crystallographic information; 3D view; checkCIF report

## Figures and Tables

**Table 1:** Hydrogen-bond geometry (Å, °)

*D*—H⋯*A*	*D*—H	H⋯*A*	*D*⋯*A*	*D*—H⋯*A*
N1—H22⋯O5^i^	0.87	2.02	2.8776 (14)	166

Symmetry code: (i) 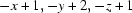.

**Fig. 1.**
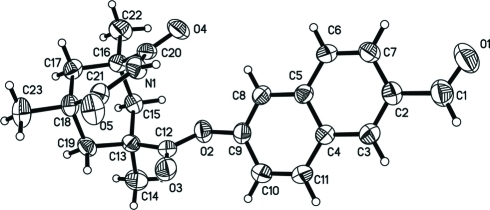
Displacement ellipsoid plot (50% probability level) of (I).

**Fig. 2.**
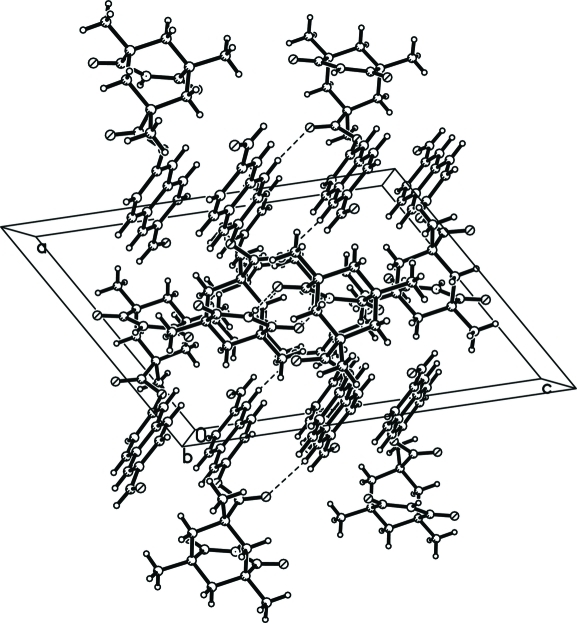
The packing diagram of (I) (viewed down the b axis)

**Fig. 3.**
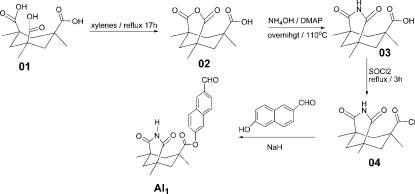
The formation of the title compound.

## supplementary crystallographic information

### Comment

The title compound (6-formylnaphthalen-2-yl *cis*-1,5,7-trimethyl-2,4-dioxo-3-aza-bicyclo[3.3.1]nonane-7-carboxylate), reported by Xu and Giuseppone (Xu *et al.*, 2008) as a key building block in a dynamic combinatorial library in which one product was self-amplified *via* self-complementary process, contains a steric bulk and a imide functional group which is a good receptor for adenine through the hydrogen bonding interaction like a base pairing. Herein we report the molecular and crystal structure of the title compound.

The unit structure of the title compound is illustrated in Fig. 1. The asymmetry of the title compound contains two ten-member rings: a naphthalene ring and a V conformation ring, which are connected each other *via *the carboxylate (O_2_—C_12_—O_3_). The naphth ring is approximately planar. In addition, the molecures are linked by the N_1_—H···O_5_ hrdrogen bond, forming inversion dimers.

### Experimental

As delineated in Scheme 1, the product was synthesized (Rebek *et al.*, 1987; Askew *et al.*, 1989; Williams *et al.*, 1989) from the starting material Kemp's triacid (01) which is commercially available. Firstly, anhydride acid (02) was prepared after dehydration of Kemp Triacid (01) under reflux 17 h in xylenes under N_2_. After anhydride acid (02) was treated with concentrated aqueous ammonium hydroxide (NH_4_OH) and 4-(dimthylamino)pyridine (DMAP) (Steglich *et al.*, 1969) at 383 K overnight, Imide Acid (03) was gained. Then we got Imide Acid Chloride (04) after the solution of Imide Acid (03) in fresh thionylchloride was reflux under nitrogen atmosphere for 3 h. At the end the target Materiel Al_1_ was prepared from Imide Acid Chloride (04) reacted with *6*-hydroxy-*2*-naphthaldehyde (Chamontin *et al.*, 1999) catalyzed by sodium hydride (NaH).

Product, yielding 60%. ^1^H NMR (400MH*_z_*,CDCl_3_, TMS) δ 10.069 (s, 1H), δ 8.244 (s, 1H), δ 7.930–7.818 (m, 3H), δ 7.589 (s, 1H), δ 7.589 (s, 1H), δ 7.540–7.535 (d, 1H), δ 7.234–7.206 (m, 1H), δ 2.822–2.787 (d, 2H), δ 2.029–1.996 (d, 1H), δ 1.488–1.267 (m, 12H); ^13^C NMR (CDCl_3_) δ 191.97, 176.06, 173.75,150.71, 137.03, 134.04, 133.97, 131.06, 130.68, 128.88, 123.60, 122.10, 118.76,44.329, 43.799, 42.625, 40.178, 30.529, 24.313; ESI-TOF Eaxct Mass for C_23_H_23_NO_5 _[*M*+Li]^+^: calcd 399.1722, 400.1731, 401.1764, 402.1791, found 399.1740, 400.1769, 401.1801, 402.1832.

### Refinement

All H atoms were positioned in calculated positions,with N—H distance of

0.86 Å, C—H distances of 0.96 Å (methyl), 0.97Å (methylene), and with

*U*iso~(H) =1.2 or 1.5 *U*eq(C, N).

### Crystal data

**Table:**

C_23_H_23_NO_5_	*F*(000) = 832
*M**_r_* = 393.42	*D*_x_ = 1.319 Mg m^−^^3^
Monoclinic, *P*2_1_/*c*	Mo *K*α radiation, λ = 0.71073 Å
Hall symbol: -P 2ybc	Cell parameters from 4643 reflections
*a* = 11.896 (2) Å	θ = 2.5–27.6°
*b* = 11.692 (2) Å	µ = 0.09 mm^−^^1^
*c* = 16.636 (2) Å	*T* = 298 K
β = 121.124 (9)°	Block, colourless
*V* = 1980.8 (5) Å^3^	0.28 × 0.20 × 0.20 mm
*Z* = 4	

### Data collection

**Table:**

Bruker APEXII CCD diffractometer	3897 independent reflections
Radiation source: fine-focus sealed tube	3217 reflections with *I* > 2σ(*I*)
graphite	*R*_int_ = 0.018
φ and ω scan	θ_max_ = 26.0°, θ_min_ = 2.0°
Absorption correction: multi-scan (*SADABS*; Bruker, 2004)	*h* = −11→14
*T*_min_ = 0.974, *T*_max_ = 0.982	*k* = −14→14
10443 measured reflections	*l* = −20→20

### Refinement

**Table:**

Refinement on *F*^2^	Primary atom site location: structure-invariant direct methods
Least-squares matrix: full	Secondary atom site location: difference Fourier map
*R*[*F*^2^ > 2σ(*F*^2^)] = 0.038	Hydrogen site location: inferred from neighbouring sites
*wR*(*F*^2^) = 0.105	H-atom parameters constrained
*S* = 1.03	*w* = 1/[σ^2^(*F*_o_^2^) + (0.0518*P*)^2^ + 0.4083*P*] where *P* = (*F*_o_^2^ + 2*F*_c_^2^)/3
3897 reflections	(Δ/σ)_max_ < 0.001
265 parameters	Δρ_max_ = 0.19 e Å^−^^3^
0 restraints	Δρ_min_ = −0.21 e Å^−^^3^

### Special details

**Table:**

Geometry. All e.s.d.'s (except the e.s.d. in the dihedral angle between two l.s. planes) are estimated using the full covariance matrix. The cell e.s.d.'s are taken into account individually in the estimation of e.s.d.'s in distances, angles and torsion angles; correlations between e.s.d.'s in cell parameters are only used when they are defined by crystal symmetry. An approximate (isotropic) treatment of cell e.s.d.'s is used for estimating e.s.d.'s involving l.s. planes.
Refinement. Refinement of *F*^2^ against ALL reflections. The weighted *R*-factor *wR* and goodness of fit *S* are based on *F*^2^, conventional *R*-factors *R* are based on *F*, with *F* set to zero for negative *F*^2^. The threshold expression of *F*^2^ > σ(*F*^2^) is used only for calculating *R*-factors(gt) *etc*. and is not relevant to the choice of reflections for refinement. *R*-factors based on *F*^2^ are statistically about twice as large as those based on *F*, and *R*– factors based on ALL data will be even larger.

### Fractional atomic coordinates and isotropic or equivalent isotropic displacement parameters (Å^2^)

**Table:**

	*x*	*y*	*z*	*U*_iso_*/*U*_eq_	
O1	1.13914 (15)	1.52850 (10)	0.66763 (9)	0.0827 (4)	
O2	0.79931 (9)	0.84989 (8)	0.48814 (7)	0.0446 (2)	
O3	0.71422 (10)	0.81424 (10)	0.57896 (7)	0.0535 (3)	
O4	0.56292 (11)	0.98673 (8)	0.30018 (7)	0.0501 (3)	
O5	0.40949 (11)	0.87877 (9)	0.48523 (8)	0.0535 (3)	
N1	0.48608 (11)	0.92723 (9)	0.39106 (8)	0.0393 (3)	
H22	0.5094	0.9926	0.4202	0.047*	
C1	1.14643 (17)	1.43589 (14)	0.70153 (12)	0.0571 (4)	
H1	1.2061	1.4268	0.7654	0.069*	
C2	1.06708 (14)	1.33579 (12)	0.64863 (10)	0.0419 (3)	
C3	1.08892 (14)	1.23220 (12)	0.69289 (10)	0.0434 (3)	
H3	1.1515	1.2268	0.7566	0.052*	
C4	1.01832 (12)	1.13362 (11)	0.64376 (9)	0.0382 (3)	
C5	0.92258 (12)	1.14345 (11)	0.54671 (9)	0.0354 (3)	
C6	0.90366 (13)	1.25141 (12)	0.50278 (10)	0.0434 (3)	
H6	0.8427	1.2584	0.4389	0.052*	
C7	0.97267 (14)	1.34465 (12)	0.55196 (10)	0.0442 (3)	
H7	0.9577	1.4149	0.5218	0.053*	
C8	0.84911 (12)	1.04604 (12)	0.49738 (9)	0.0393 (3)	
H8	0.7853	1.0515	0.4340	0.047*	
C9	0.87159 (13)	0.94461 (12)	0.54251 (9)	0.0393 (3)	
C10	0.96829 (15)	0.93151 (13)	0.63736 (10)	0.0500 (4)	
H10	0.9842	0.8604	0.6663	0.060*	
C11	1.03874 (15)	1.02504 (13)	0.68656 (10)	0.0509 (4)	
H11	1.1019	1.0172	0.7499	0.061*	
C12	0.72086 (12)	0.79239 (11)	0.51122 (9)	0.0363 (3)	
C13	0.65192 (13)	0.69261 (10)	0.44424 (9)	0.0362 (3)	
C14	0.75342 (16)	0.59438 (13)	0.48707 (12)	0.0572 (4)	
H14A	0.7185	0.5273	0.4486	0.086*	
H14B	0.7718	0.5780	0.5493	0.086*	
H14C	0.8330	0.6169	0.4899	0.086*	
C15	0.61823 (13)	0.71551 (11)	0.34325 (9)	0.0369 (3)	
H15A	0.6067	0.6425	0.3123	0.044*	
H15B	0.6928	0.7532	0.3459	0.044*	
C16	0.49451 (13)	0.78899 (10)	0.28177 (8)	0.0359 (3)	
C17	0.37796 (13)	0.73661 (11)	0.28312 (9)	0.0382 (3)	
H17A	0.3616	0.6602	0.2567	0.046*	
H17B	0.3001	0.7824	0.2447	0.046*	
C18	0.40466 (13)	0.73080 (10)	0.38319 (9)	0.0360 (3)	
C19	0.52754 (13)	0.65756 (11)	0.44435 (9)	0.0389 (3)	
H19A	0.5468	0.6601	0.5085	0.047*	
H19B	0.5071	0.5789	0.4233	0.047*	
C20	0.51756 (13)	0.90892 (10)	0.32291 (8)	0.0358 (3)	
C21	0.43114 (13)	0.85050 (11)	0.42433 (9)	0.0374 (3)	
C22	0.47044 (18)	0.79546 (14)	0.18231 (10)	0.0546 (4)	
H22A	0.3959	0.8434	0.1443	0.082*	
H22B	0.4539	0.7201	0.1557	0.082*	
H22C	0.5465	0.8269	0.1844	0.082*	
C23	0.28802 (16)	0.68016 (14)	0.38570 (12)	0.0558 (4)	
H23A	0.3061	0.6813	0.4490	0.084*	
H23B	0.2740	0.6027	0.3633	0.084*	
H23C	0.2108	0.7247	0.3463	0.084*	

### Atomic displacement parameters (Å^2^)

**Table:**

	*U*^11^	*U*^22^	*U*^33^	*U*^12^	*U*^13^	*U*^23^
O1	0.1151 (11)	0.0470 (7)	0.0734 (9)	−0.0257 (7)	0.0398 (8)	−0.0030 (6)
O2	0.0485 (6)	0.0427 (5)	0.0449 (5)	−0.0134 (4)	0.0257 (5)	−0.0075 (4)
O3	0.0559 (6)	0.0680 (7)	0.0351 (5)	−0.0186 (5)	0.0224 (5)	−0.0079 (5)
O4	0.0676 (7)	0.0338 (5)	0.0467 (6)	−0.0063 (5)	0.0280 (5)	0.0051 (4)
O5	0.0652 (7)	0.0502 (6)	0.0614 (7)	−0.0137 (5)	0.0441 (6)	−0.0232 (5)
N1	0.0515 (7)	0.0253 (5)	0.0403 (6)	−0.0041 (5)	0.0232 (5)	−0.0089 (4)
C1	0.0697 (10)	0.0489 (9)	0.0525 (9)	−0.0152 (8)	0.0315 (8)	−0.0069 (7)
C2	0.0436 (7)	0.0406 (7)	0.0456 (8)	−0.0052 (6)	0.0258 (6)	−0.0029 (6)
C3	0.0428 (7)	0.0473 (8)	0.0339 (7)	−0.0055 (6)	0.0155 (6)	−0.0003 (6)
C4	0.0345 (6)	0.0408 (7)	0.0354 (7)	−0.0020 (5)	0.0153 (6)	0.0017 (5)
C5	0.0294 (6)	0.0405 (7)	0.0348 (7)	0.0003 (5)	0.0155 (5)	0.0020 (5)
C6	0.0354 (7)	0.0472 (8)	0.0378 (7)	0.0017 (6)	0.0120 (6)	0.0091 (6)
C7	0.0413 (7)	0.0381 (7)	0.0512 (8)	0.0016 (6)	0.0226 (7)	0.0088 (6)
C8	0.0334 (6)	0.0463 (8)	0.0329 (7)	−0.0039 (6)	0.0133 (5)	0.0013 (6)
C9	0.0367 (7)	0.0406 (7)	0.0400 (7)	−0.0073 (6)	0.0194 (6)	−0.0045 (6)
C10	0.0502 (8)	0.0401 (8)	0.0460 (8)	−0.0035 (6)	0.0150 (7)	0.0094 (6)
C11	0.0486 (8)	0.0484 (8)	0.0344 (7)	−0.0061 (7)	0.0062 (6)	0.0068 (6)
C12	0.0347 (6)	0.0360 (7)	0.0320 (6)	−0.0002 (5)	0.0129 (5)	0.0054 (5)
C13	0.0392 (7)	0.0280 (6)	0.0387 (7)	0.0009 (5)	0.0182 (6)	0.0028 (5)
C14	0.0540 (9)	0.0398 (8)	0.0697 (11)	0.0108 (7)	0.0262 (8)	0.0125 (7)
C15	0.0438 (7)	0.0304 (6)	0.0418 (7)	−0.0015 (5)	0.0259 (6)	−0.0060 (5)
C16	0.0458 (7)	0.0309 (6)	0.0289 (6)	−0.0017 (5)	0.0179 (6)	−0.0042 (5)
C17	0.0394 (7)	0.0328 (6)	0.0356 (7)	−0.0032 (5)	0.0146 (6)	−0.0097 (5)
C18	0.0382 (7)	0.0320 (6)	0.0394 (7)	−0.0071 (5)	0.0212 (6)	−0.0095 (5)
C19	0.0484 (8)	0.0291 (6)	0.0396 (7)	−0.0067 (5)	0.0229 (6)	0.0000 (5)
C20	0.0409 (7)	0.0285 (6)	0.0294 (6)	0.0013 (5)	0.0121 (5)	0.0024 (5)
C21	0.0368 (7)	0.0350 (7)	0.0398 (7)	−0.0035 (5)	0.0192 (6)	−0.0096 (5)
C22	0.0745 (11)	0.0542 (9)	0.0339 (7)	−0.0095 (8)	0.0272 (7)	−0.0063 (7)
C23	0.0519 (9)	0.0570 (9)	0.0674 (10)	−0.0215 (7)	0.0370 (8)	−0.0208 (8)

### Geometric parameters (Å, °)

**Table:**

O1—C1	1.2031 (19)	C12—C13	1.5267 (18)
O2—C12	1.3569 (15)	C13—C15	1.5358 (18)
O2—C9	1.4071 (16)	C13—C19	1.5363 (18)
O3—C12	1.1971 (16)	C13—C14	1.5477 (19)
O4—C20	1.2136 (15)	C14—H14A	0.9600
O5—C21	1.2132 (15)	C14—H14B	0.9600
N1—C21	1.3828 (17)	C14—H14C	0.9600
N1—C20	1.3822 (17)	C15—C16	1.5473 (18)
N1—H22	0.8701	C15—H15A	0.9700
C1—C2	1.475 (2)	C15—H15B	0.9700
C1—H1	0.9300	C16—C20	1.5218 (17)
C2—C3	1.3707 (19)	C16—C17	1.5264 (19)
C2—C7	1.411 (2)	C16—C22	1.5284 (18)
C3—C4	1.4115 (19)	C17—C18	1.5266 (18)
C3—H3	0.9300	C17—H17A	0.9700
C4—C11	1.4138 (19)	C17—H17B	0.9700
C4—C5	1.4199 (18)	C18—C21	1.5177 (17)
C5—C8	1.4118 (18)	C18—C23	1.5289 (19)
C5—C6	1.4169 (18)	C18—C19	1.5388 (19)
C6—C7	1.356 (2)	C19—H19A	0.9700
C6—H6	0.9300	C19—H19B	0.9700
C7—H7	0.9300	C22—H22A	0.9600
C8—C9	1.3542 (19)	C22—H22B	0.9600
C8—H8	0.9300	C22—H22C	0.9600
C9—C10	1.400 (2)	C23—H23A	0.9600
C10—C11	1.363 (2)	C23—H23B	0.9600
C10—H10	0.9300	C23—H23C	0.9600
C11—H11	0.9300		
			
C12—O2—C9	119.29 (10)	H14A—C14—H14C	109.5
C21—N1—C20	127.75 (10)	H14B—C14—H14C	109.5
C21—N1—H22	115.3	C13—C15—C16	116.17 (10)
C20—N1—H22	116.7	C13—C15—H15A	108.2
O1—C1—C2	124.44 (16)	C16—C15—H15A	108.2
O1—C1—H1	117.8	C13—C15—H15B	108.2
C2—C1—H1	117.8	C16—C15—H15B	108.2
C3—C2—C7	119.69 (13)	H15A—C15—H15B	107.4
C3—C2—C1	119.70 (13)	C20—C16—C17	108.48 (10)
C7—C2—C1	120.56 (13)	C20—C16—C22	109.31 (11)
C2—C3—C4	121.31 (13)	C17—C16—C22	111.87 (11)
C2—C3—H3	119.3	C20—C16—C15	109.00 (10)
C4—C3—H3	119.3	C17—C16—C15	109.17 (10)
C3—C4—C11	123.08 (12)	C22—C16—C15	108.96 (11)
C3—C4—C5	118.67 (12)	C16—C17—C18	110.81 (10)
C11—C4—C5	118.26 (12)	C16—C17—H17A	109.5
C8—C5—C6	122.23 (12)	C18—C17—H17A	109.5
C8—C5—C4	119.16 (12)	C16—C17—H17B	109.5
C6—C5—C4	118.61 (12)	C18—C17—H17B	109.5
C7—C6—C5	121.33 (12)	H17A—C17—H17B	108.1
C7—C6—H6	119.3	C21—C18—C17	109.17 (10)
C5—C6—H6	119.3	C21—C18—C23	108.94 (11)
C6—C7—C2	120.38 (12)	C17—C18—C23	111.52 (11)
C6—C7—H7	119.8	C21—C18—C19	108.07 (10)
C2—C7—H7	119.8	C17—C18—C19	109.68 (10)
C9—C8—C5	119.91 (12)	C23—C18—C19	109.38 (12)
C9—C8—H8	120.0	C13—C19—C18	115.92 (10)
C5—C8—H8	120.0	C13—C19—H19A	108.3
C8—C9—C10	122.17 (12)	C18—C19—H19A	108.3
C8—C9—O2	116.93 (12)	C13—C19—H19B	108.3
C10—C9—O2	120.76 (12)	C18—C19—H19B	108.3
C11—C10—C9	118.73 (13)	H19A—C19—H19B	107.4
C11—C10—H10	120.6	O4—C20—N1	119.48 (11)
C9—C10—H10	120.6	O4—C20—C16	123.81 (12)
C10—C11—C4	121.71 (13)	N1—C20—C16	116.71 (10)
C10—C11—H11	119.1	O5—C21—N1	120.38 (11)
C4—C11—H11	119.1	O5—C21—C18	122.93 (12)
O3—C12—O2	123.39 (12)	N1—C21—C18	116.66 (11)
O3—C12—C13	125.33 (12)	C16—C22—H22A	109.5
O2—C12—C13	111.13 (11)	C16—C22—H22B	109.5
C12—C13—C15	113.79 (10)	H22A—C22—H22B	109.5
C12—C13—C19	110.93 (10)	C16—C22—H22C	109.5
C15—C13—C19	109.93 (11)	H22A—C22—H22C	109.5
C12—C13—C14	103.54 (11)	H22B—C22—H22C	109.5
C15—C13—C14	109.47 (11)	C18—C23—H23A	109.5
C19—C13—C14	108.91 (11)	C18—C23—H23B	109.5
C13—C14—H14A	109.5	H23A—C23—H23B	109.5
C13—C14—H14B	109.5	C18—C23—H23C	109.5
H14A—C14—H14B	109.5	H23A—C23—H23C	109.5
C13—C14—H14C	109.5	H23B—C23—H23C	109.5
			
O1—C1—C2—C3	175.94 (17)	C12—C13—C15—C16	−79.40 (14)
O1—C1—C2—C7	−1.4 (3)	C19—C13—C15—C16	45.71 (14)
C7—C2—C3—C4	−0.3 (2)	C14—C13—C15—C16	165.31 (11)
C1—C2—C3—C4	−177.65 (13)	C13—C15—C16—C20	64.94 (14)
C2—C3—C4—C11	179.38 (14)	C13—C15—C16—C17	−53.40 (14)
C2—C3—C4—C5	−0.3 (2)	C13—C15—C16—C22	−175.85 (11)
C3—C4—C5—C8	−178.46 (12)	C20—C16—C17—C18	−59.52 (13)
C11—C4—C5—C8	1.79 (19)	C22—C16—C17—C18	179.83 (11)
C3—C4—C5—C6	1.19 (19)	C15—C16—C17—C18	59.14 (13)
C11—C4—C5—C6	−178.56 (13)	C16—C17—C18—C21	58.72 (13)
C8—C5—C6—C7	178.15 (13)	C16—C17—C18—C23	179.15 (11)
C4—C5—C6—C7	−1.5 (2)	C16—C17—C18—C19	−59.53 (13)
C5—C6—C7—C2	0.9 (2)	C12—C13—C19—C18	81.06 (13)
C3—C2—C7—C6	0.0 (2)	C15—C13—C19—C18	−45.66 (14)
C1—C2—C7—C6	177.36 (13)	C14—C13—C19—C18	−165.61 (12)
C6—C5—C8—C9	179.57 (13)	C21—C18—C19—C13	−65.35 (14)
C4—C5—C8—C9	−0.79 (19)	C17—C18—C19—C13	53.57 (14)
C5—C8—C9—C10	−1.3 (2)	C23—C18—C19—C13	176.18 (11)
C5—C8—C9—O2	−177.05 (11)	C21—N1—C20—O4	178.38 (13)
C12—O2—C9—C8	−121.10 (13)	C21—N1—C20—C16	−2.65 (19)
C12—O2—C9—C10	63.13 (17)	C17—C16—C20—O4	−150.00 (13)
C8—C9—C10—C11	2.4 (2)	C22—C16—C20—O4	−27.77 (19)
O2—C9—C10—C11	177.95 (13)	C15—C16—C20—O4	91.23 (15)
C9—C10—C11—C4	−1.3 (2)	C17—C16—C20—N1	31.08 (15)
C3—C4—C11—C10	179.53 (15)	C22—C16—C20—N1	153.30 (12)
C5—C4—C11—C10	−0.7 (2)	C15—C16—C20—N1	−87.70 (14)
C9—O2—C12—O3	−3.89 (19)	C20—N1—C21—O5	179.58 (13)
C9—O2—C12—C13	−179.69 (11)	C20—N1—C21—C18	1.5 (2)
O3—C12—C13—C15	150.90 (13)	C17—C18—C21—O5	152.91 (13)
O2—C12—C13—C15	−33.39 (14)	C23—C18—C21—O5	30.91 (18)
O3—C12—C13—C19	26.33 (18)	C19—C18—C21—O5	−87.84 (15)
O2—C12—C13—C19	−157.96 (10)	C17—C18—C21—N1	−29.08 (16)
O3—C12—C13—C14	−90.35 (16)	C23—C18—C21—N1	−151.08 (13)
O2—C12—C13—C14	85.35 (13)	C19—C18—C21—N1	90.17 (14)

### Hydrogen-bond geometry (Å, °)

**Table:**

*D*—H···*A*	*D*—H	H···*A*	*D*···*A*	*D*—H···*A*
N1—H22···O5^i^	0.87	2.02	2.8776 (14)	166

Symmetry codes: (i) −*x*+1, −*y*+2, −*z*+1.
